# Insights into Macrophage Heterogeneity and Cytokine-Induced Neuroinflammation in Major Depressive Disorder

**DOI:** 10.3390/ph11030064

**Published:** 2018-06-25

**Authors:** Adwitia Dey, Pamela A. Hankey Giblin

**Affiliations:** 1 Center for Molecular Immunology and Infectious Diseases, The Pennsylvania State University, University Park, State College, PA 16802, USA; phc7@psu.edu; 2 Department of Veterinary and Biomedical Sciences, The Pennsylvania State University, University Park, State College, PA 16802, USA

**Keywords:** depression, macrophages, cytokine-mediated inflammation

## Abstract

Over 350 million individuals suffer from depression, a psychiatric illness classified as major depressive disorder (MDD) with symptoms that include a loss of interest or pleasure in life accompanied by depressed mood. The present understanding of major depressive disorder does not encompass a systematic characterization of the neurobiological processes that drive the behavioral physiology in patients diagnosed with major depressive disorder. Psychiatric illness is a complex intersection between genetics, physiology, immunology and environmental stress. The increased attention to the relevance of depression has led to new discoveries that highlight the biological significance of ‘neuroinflammation’ and immunity underlying a spectrum of psychiatric illnesses. The process of neuroinflammation involves sentinel immune cells in the central nervous system (CNS). The activation and polarization of microglia, CNS-resident macrophages, modulates the production and secretion of pro-inflammatory cytokines implicated in the etiology of major depressive disorder, and this phenomenon has been aptly titled the ‘macrophage theory of depression’. Of particular interest are three hallmark cytokines, IL-6, TNFα and IL-1β, which have been studied extensively in basic research, cell-receptor signaling and drug development. The field of inflammasome-mediated neuroinflammation is an emerging area of MDD research that is providing new cellular insight into how macrophages mechanistically support cytokine-associated neuropathology, particularly in the case of IL-1β-associated inflammation in MDD. With the increasing number of individuals identified with depression, a comprehensive understanding of macrophage-cytokine signaling pathways in the CNS in depression is necessary for developing effective anti-depressant therapeutics.

## 1. Introduction

For centuries, civilization separated mind from matter. Hippocrates of Kos was the ancient Greek physician who described symptoms of melancholy as a disease state with physical and mental symptoms. In past centuries, prominent authors and psychiatrists developed the metaphorical and physiological understanding of depression as a lessening of emotional strength. However, it was not until the early 20th Century that scientists began to understand the biological foundation of this illness. With the growing epidemic of depression and increasing lifetime prevalence, the blurred lines between mind and matter have become an intersecting point of study for neuroscientists in the 21st Century. Nearly 350 million individuals globally suffer from depression, a psychiatric illness classified in the 1970s as major depressive disorder with symptoms that encompass a loss of interest or pleasure in life and depressed mood [1]. Though depression has been prevalent from the dawn of man, it was not until the beginning of the 20th Century that societies started to acknowledge the biological consequences and economic toll of depression on civilization. In 2001, the World Health Organization (WHO) published a mental health report that established depression as a major source of disability [2,3]. However, the present understanding of major depressive disorder (MDD) does not encompass thorough characterization of neurobiological processes that drive the behavioral physiology found in patients diagnosed with MDD.

In the U.S. alone, half of the population will exhibit a psychiatric illness in their lifetime, with depression being the most prevalent. Recent figures indicate that depression in the U.S. is linked to significantly decreased workplace productivity that is costing the U.S. economy at least $30 billion per year [4]. The global incidence of psychiatric disorders such as MDD has increased to a critical point, and the WHO projects depression will be the leading cause of disease burden by 2030 [5]. Psychiatric illnesses intersect the understanding of immunological processes with genetic and environmental stress. With physiological stressors accruing in the global climate, more and more psychiatric disorders are being identified and acknowledged. Meanwhile, the neurological mechanisms underlying MDD have not been well characterized. The increased attention to the relevance of depression has led to new discoveries that highlight the biological significance of ‘neuroinflammation’ underlying a spectrum of psychiatric illnesses [6]. Understanding the biological underpinnings of neuroinflammation can provide valuable insight into how psychiatric disorders, in particular depression, develop and manifest at a cellular level, so that we can begin to decipher and appreciate the pharmacological mechanisms behind this growing global health crisis.

Linking neuroinflammation to psychiatric disorders was proposed decades ago, with researchers identifying a biological link between depression and underlying inflammation in the central nervous system [7]. Neuroinflammation is the activation of immune cells in the CNS and is studied in neurodegenerative diseases such as Alzheimer’s disease, Parkinson’s disease and Huntington’s disease [8,9]. It is from patients with severe neuropathology that clinicians began to tease out secondary depressive symptoms [10]. The process of neuroinflammation involves sentinel immune cells in the CNS, resident macrophages known as microglia. Research in this field has alluded to a ‘macrophage theory of depression’, which implicates excessive and uncontrolled activation of macrophage-associated cytokines in the neuropathology underlying the MDD disease state [11]. Since then, more and more evidence for macrophage-associated inflammation has been linked to the pathophysiology underlying MDD. Microglia are a unique group of CNS macrophages that are quiescent surveillance cells, ‘inducible’ upon a stress stimulus and can be subsequently activated to secrete inflammatory cytokines [12]. The CNS proper is sustained upon a network of various cell types such as astrocytes, oligodendrocytes and neurons; however, microglia are the prime immune responders to stressors, irritants and pathogens [13]. Prolonged environmental stress stimulates continuous cytokine secretion, which leads to uncontrolled inflammation in the CNS proper. This theory of depression implicates both arms of the immune network, innate and adaptive. In this review, we will focus on studies describing the contribution of the innate immune system. We will review current progress in understanding the relationship between macrophage-associated cytokine-induced inflammation and inflammasome activation underlying MDD.

## 2. Macrophage Heterogeneity in Neuroinflammation and Major Depressive Disorder

Macrophages are innate immune cells that maintain homeostasis by governing the induction and resolution of inflammation. In recent years, researchers have identified a critical role for macrophage heterogeneity and polarization in neuronal health and disease [14]. Here, we focus on tissue-resident macrophages, with some attention to circulating monocytes and trafficked macrophages. Macrophages are sentinel immune cells present in all tissues that detect infection or stressors in the tissue microenvironment. In addition to resident tissue macrophages, monocytes from the circulation can be recruited to sites of infection or inflammation to further promote innate and adaptive immunity [15]. Macrophages express a variety of pattern recognition receptors (PRRs), present at the cell surface, within endosomal compartments and in the cytosol, which recognize broadly-conserved patterns on invading pathogens called pattern-associated molecular patterns (PAMPs), as well as danger-associated molecular patterns (DAMPs) that are generated in response to cellular stress [16]. In response to these triggers, pattern recognition receptors upregulate the central inflammatory transcription factor NFκB, which orchestrates the rapid upregulation of an array of pro-inflammatory cytokines [17]. While this response plays a critical role in the clearance of pathogens and initiating repair mechanisms in response to damage, when uncontrolled, pro-inflammatory macrophage activation results in chronic inflammation that can promote both local and systemic tissue damage. 

Macrophages are broadly characterized as classically-activated (M1) cells, which secrete pro-inflammatory cytokines, or as alternatively-activated (M2) cells, which foster tissue repair and regeneration [18,19]. Biologically, these two phenotypes are typified by pathways regulating arginine metabolism. The expression of inducible nitric oxide synthase (iNOS) is upregulated in M1 macrophages and converts l-arginine to nitric oxide (NO), cytotoxic levels of which can promote tissue injury. Conversely, arginase-1 (Arg-1), upregulated in M2 macrophages, metabolizes L-arginine to l-citrulline, resulting in the generation of polyamines and prolines, factors that promote tissue repair, proliferation and matrix synthesis [19]. In addition to the production of pro-inflammatory cytokines, M1-macrophages exhibit more potent phagocytic activity and increased microbicidal activity. The M1-phenotype, induced by pathogen-associated molecular patterns including peptidoglycans, lipopolysaccharide (LPS) or flagellin present on invading pathogens, results in the expression of inflammatory cytokines including interleukin 1β (IL-1β), interleukin 12 (IL-12) and tumor necrosis factor-α (TNFα). IL-12, in turn, induces T helper 1 (Th1) cell development, which promotes a positive feedback loop through the secretion of high levels of interferon gamma (IFNγ), which cooperates with PRRs to perpetuate the M1 macrophage phenotype [18]. Alternatively, the M2 macrophage phenotype is induced by the T helper 2 (Th2) cytokines IL-4 and IL-13, as well as the anti-inflammatory cytokines IL-10 and transforming growth factor beta (TGFβ) or immune suppressing glucocorticoid hormones [20,21]. In addition to the upregulation of Arg-1 and tissue repair functions, M2-macrophage activation promotes the upregulation of IL-10 and TGFβ, which in turn, provide feedback to further suppress inflammatory M1-macrophage activation. 

M1 and M2 macrophages are likely the polar ends of a spectrum of complex macrophage phenotypes [22,23]. Some classifications expand the M2 subpopulation into distinct groups depending on the mechanism of activation in vitro. Wound-healing or M2a macrophages represent the originally-identified M2 population induced by IL-4 and IL-13, while regulatory or M2b macrophages are induced by immune complexes. M2c macrophages have also been described based on their polarization in vitro by IL-10 or glucocorticoids. These have also been characterized as M0 or downregulated macrophages. However, it remains unclear whether these phenotypes induced in vitro represent the spectrum of macrophages found in vivo. Many more subpopulations of macrophages have been described based on their presence in specific disease states like atherosclerosis [24]. Here, for simplicity, we use the M1/M2 paradigm where M2 macrophages would encompass all of the spectrum of macrophages with downregulated, regulatory or wound-healing phenotypes.

In addition to a spectrum of macrophage phenotypes that can be induced by environmental cues, tissue-resident macrophages exhibit a wide range of functions, depending on the tissue microenvironment, in order to promote tissue homeostasis [25]. Even within a given tissue, multiple distinct populations of resident macrophages can exist, depending on the anatomical location within the tissue parenchyma [26]. In the CNS, there exists four distinct macrophage populations that are distinguished by their function and cell surface antigens [27]. The majority of macrophages in the CNS are microglia, which express CD45^low^F480^+^CD11B^+^ surface antigens and are localized throughout the parenchyma [26,28]. Microglia are an ontogenically unique yolk-sac-derived population of cells and play a critical role in CNS homeostasis and disease [13]. Erythromyeloid progenitor cells from the yolk sac seed the CNS during embryonic development and differentiate into microglia, prior to the formation of the blood-brain-barrier (BBB) [25,29]. These progenitors arise before the development of hematopoietic stem cells; thus, microglia arise independent of hematopoietic stem cells and constitute a distinct ontological lineage. These CNS-resident microglia then persist for life, dividing only infrequently to maintain their numbers [30]. Like peripheral macrophages, microglia have also been shown to adopt M1 and M2 phenotypes.

During inflammation, peripheral monocytes can be recruited to the CNS. However, while infiltrating monocytes have been shown to trigger disease progression in the CNS, they are short-lived and do not contribute to the resident microglial pool [31]. The other three surveillance macrophage populations in the CNS are perivascular, meningeal and choroid plexus macrophages. These cells are characterized by the expression of CD45^high^F480^+^CD11B^+^ surface antigens and are localized at CNS-periphery tissue interfaces [12,27]. Although less well characterized than microglia, these cells facilitate the influx of leukocytes at the blood brain barrier and have also been implicated in the development, progression and resolution of inflammation in the CNS. While these macrophage populations have long been thought to be derived from monocytes, recent fate mapping studies have shown that these cells are also derived from erythromyeloid progenitors from the yolk sac. However, while maintenance of perivascular and meningeal macrophages does not depend on monocytes, choroid plexus macrophages are continually replenished by peripheral monocytes [32].

**Figure 1:**
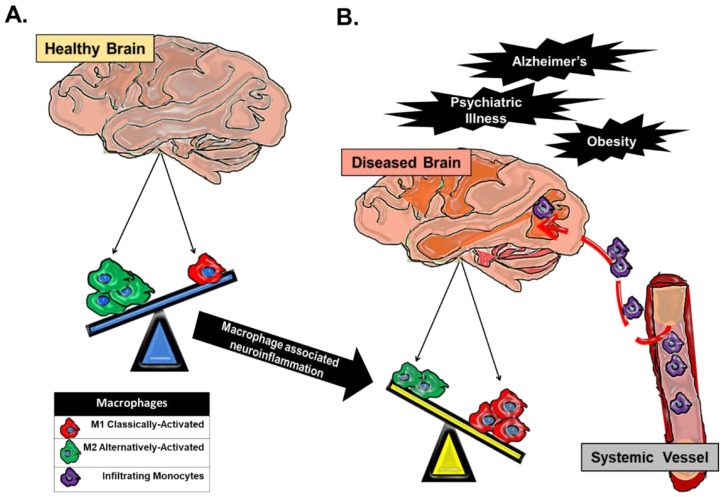
Macrophage dynamics in CNS health and disease. Macrophages in the CNS can be broadly categorized as classically-activated (M1) macrophages or alternatively-activated (M2) macrophages. (**A**) CNS tissue-resident macrophages are microglial cells, and in a homeostatic healthy state, a predominance of CNS microglia are found in a quiescent M2 state. (**B**) In any CNS disease state that has an underlying component of inflammation, there is a skewing of macrophage populations towards an M1 phenotype. This skewing can be attributed to an alteration in the balance of tissue-resident macrophage activation away from an M2 phenotype towards an M1 phenotype, which propagates pro-inflammatory cytokine secretion in the CNS parenchyma. Chronic inflammation can perpetuate the breakdown of the ‘immuno-privileged’ blood brain barrier and allow for trafficked monocytes to enter the CNS proper. Trafficked monocytes become activated M1 macrophages in response to signals in the CNS microenvironment and contribute to the existing inflammation.

During development and in the adult, microglia and neurons have intimate interactions that are essential for synaptic pruning, health and behavior [33]. In healthy CNS tissue, microglia are in a quiescent surveillance state characterized by a downregulated M2 phenotype, which is critical for neuronal growth and homeostatic neuron-glial interactions [25]. This interaction is mediated by chemokine CX3C chemokine ligand 1 (CX3CL1) and the membrane protein, CD200, expressed on neurons that interact with the microglial receptors CX3CR1 and CD200R, respectively [34,35,36]. CD200R carries immunoreceptor tyrosine-based inhibitory motifs (ITIMs) such that, upon ligand-receptor interaction, the ITIM-based receptors suppress downstream inflammatory immune signaling through the recruitment of Src homology 2 domain-containing phosphatase 1 (SHP-1), which negatively regulates signaling by PRRs [35]. Microglia also release neurotropic factors including nerve growth factor (NGF), brain-derived neurotrophic factor (BDNF) and glial-derived neurotrophic factor (GDNF), which promote axonal outgrowth and synaptogenesis. Immunologically, this M2 microglial phenotype is characterized by attenuated innate immune function correlated with decreased cell surface expression of CD45, MHCII and Fc receptors [23,37]. Physiological or environmental strain stimulates oxidative stress and the production of reactive oxygen species (ROS), which trigger the transformation of microglia from a quiescent to an activated state [14]. During this activation, microglia alter their morphology from a dendritic structure to a more amoeboid shape and diverge away from neuronal interactions that otherwise maintain the quiescent state. This activation is similar to M1 macrophages and is characterized by elevated glial levels of ionized calcium-binding adapter molecule 1 (IBA-1) and microglial secretion of the pro-inflammatory cytokines IL-1β and TNF-α [38]. In response to stress, infiltrating bone marrow-derived monocytes primarily differentiate into macrophages that are skewed toward an M1 phenotype in response to factors produced by cells in the tissue niche [39]. As clinical studies in neurodegenerative diseases such as Alzheimer’s disease have progressed, M1 macrophages have been identified as fundamental contributors to inflammation-mediated neurodegeneration and subsequent cognitive impairment (Figure 1) [38].

M1 macrophages are significant contributors to inflammation in neurodegenerative patients exhibiting severe depression [12]. Symptoms of depression in these patients have linked neuroinflammation to the monoamine hypothesis of depression, where there is a reduction in serotonin/5-hydroxytryptamine(5-HT) levels [40,41]. M1-associated cytokine activation in the CNS proper decreases 5-HT production and regulates tryptophan metabolism to quinolinic acid (QA) and kynurenic acid (KYN) [42]. Elevated M1 macrophage recruitment has been localized to postmortem dorsal anterior cingulate matter from individuals suffering from MDD [43]. In behavior-induced stress studies such as the Trier Social Stress Test (TSTT), conducted on depressed subjects, researchers have found increased levels of circulating cytokines and upregulation of the hallmark inflammatory NF-kB in peripheral blood mononuclear cells (PBMCs) in response to stress [44]. There is extensive cross-talk between the innate and adaptive arms of the immune system, which synergistically contributes to the underlying neuroinflammation. Maladaptive regulation of Th-1, Th2 and Th17 cell response leads to an increased Th-1 (M1-macrophage associated) positive feedback loop of inflammatory cytokine secretion and decreased regulatory T-cell (T-Reg) population in patients with depressive symptoms [7,45]. Altogether, balancing macrophage heterogeneity is central to regulating levels of inflammation underlying neuropathology [46]. 

## 3. Cytokines and Their Role in Major Depressive Disorder

**Figure 2:**
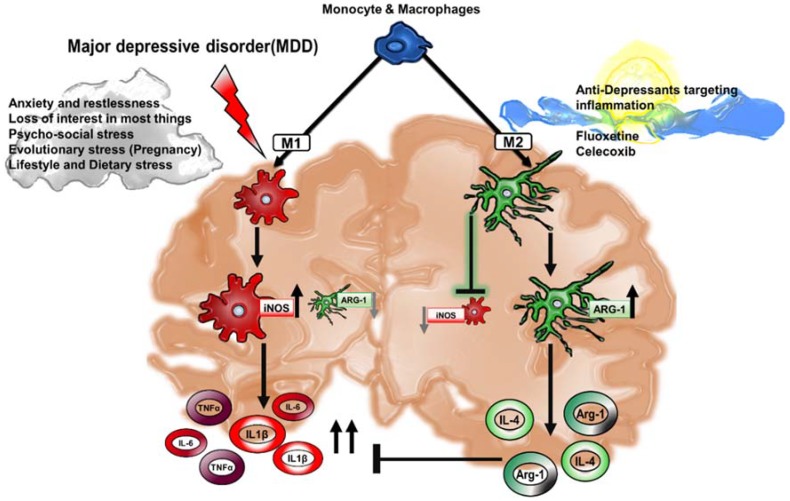
Macrophage-associated cytokines in MDD. In a state of chronic depression, excessive inflammation contributes to a positive feedback loop of pro-inflammatory cytokines in the CNS parenchyma. M1 activation in MDD corresponds to macrophage-associated secretion of pro-inflammatory cytokines interleukin-1β (IL-1β), interleukin-6 (IL-6) and tumor necrosis factor-α (TNF-α) and a decrease in the activation of anti-inflammatory M2 markers such as Arginase-1 (Arg-1). A healthy brain sustains M2 macrophages that foster the secretion of interleukin-4 (IL-4) and Arg-1, that altogether act to inhibit the uncontrolled inflammation supported by M1 macrophages. The expansion of M2 macrophages is a critical strategy that is oftentimes the signaling target of anti-depressant drugs in order to curb M1-associated injury.

Cytokines are a broad class of small proteins secreted by various cell types including macrophages [47]. They can act in an autocrine manner to impact cells that secrete them, in a paracrine manner to act on nearby/local cells or in an endocrine manner to act on distant cells [47,48]. Cells respond to cytokines in the microenvironment through a large family of cytokine receptors. In addition to the activation of NFκB in response to cytokine stimulation, cytokine receptors signal through activation of the signal transducer and activator of transcription (STAT) protein family of transcription factors to regulate the balance between M1 and M2-macrophage activation. IFNγ, produced by Th1 cells, signals through the type II IFN receptor to induce the activation of Stat1. Stat1 then cooperates with NFκB to promote M1 macrophage activation [20,21]. Alternatively, the Th2 cytokines IL-4 and IL-13 promote the activation of Stat6, which drives expression of M2-associated genes, and the anti-inflammatory cytokine, IL-10, produced by M2 macrophages, promotes Stat3 activation, which serves to inhibit the activation of M1-associated genes [20,49]. Of the pro-inflammatory cytokines, M1 macrophage-derived IL-6, TNFα and IL1β have been implicated in the progression of MDD (Figure 2) [50].

The activation of Janus kinase (JAK)-STAT signaling by IL-6 in circulating immune cells along with an increased circulating plasma indoleamine 2,3-dioxygenase (IDO) activity have been correlated to depression in patients [51]. Computational analysis of the human genome has found that a single nucleotide change in the IL-6 promoter is associated with heightened risk of neuroinflammation, particularly in populations that experience socioeconomic strife [52]. In controlled studies, minor environmental stress leads to increased levels of IL-6 in the blood and cerebrospinal fluid (CSF). Increased levels of hippocampal IL-6 were observed in murine models of depression following induction of seasonal affective disorder, whereas deletion of IL-6 resulted in a reduction of depression symptomology [53,54]. MDD is closely linked to decreased expression of neuronal trophic factors, including the glial cell line derived neurotrophic factor (GDNF), brain derived neurotrophic factor (BDNF) and nerve growth factor (NGF) [55]. Elevated IL-6 levels in MDD are associated with a decrease in BDNF expression in the CNS [55]. The peroxisome proliferator-activated receptor gamma (PPARγ) agonist from the thiazolidinediones family, pioglitazone (PIO), inhibits the IL-6 pathway in mice. Liao et al. demonstrated an improvement in depressive behaviors induced by intraventricular administration of LPS in mice, associated with a decrease in IL-6 and restoration in BDNF levels [55]. Therefore, IL-6 is implicated in acutely-derived or chronically-derived stressors of depression, and it is an important target for anti-inflammatory agonists that support M2-macrophage expansion.

Tumor necrosis factor-α (TNFα) is a soluble cytokine that was discovered approximately three decades ago by Carswell et al. as a potent anti-tumor (necrosis) agent. With recent advents in immunological research, TNFα has become a hallmark M1-macrophage-associated cytokine in the pathophysiology of chronic inflammatory disease and has been implicated in the inflammation underlying neurodegenerative diseases and aging-associated dementia, neurodevelopmental disorders and clinical depression [56]. Transgenic mice with CNS-specific TNFα overexpression exhibited severe inflammation associated with demyelination, microgliosis and astrogliosis-induced scarring [57]. Interestingly, TNFα also activates the hypothalamo-pituitary-adrenocortical (HPA) axis and subsequent depletion of tryptophan through the activation of IDO [58,59]. IDO activation leads to uncontrolled glutamatergic activation and decreased availability of serotonin, the classical target for anti-depressants [60]. Clinical studies in MDD confirm a correlation between circulating TNFα levels and the progressive development of depressive symptoms [50,56,61]. Acute treatment with the anti-depressant fluoxetine, an SSRI, reduced circulating levels of TNFα. In vitro monocyte cultures with fluoxetine treatment attenuated TNFα production in a dose-dependent manner [62]. The TNFα inhibitor, etanercept, attenuated depressive symptoms in a murine model of chronic mild stress [63]. Systemic inflammation and ‘peripheral immune challenges’ trigger anxiety-induced depressive states. Focal etanercept administration and attenuation of CNS TNFα accumulation improved anxiety-associated behavior and depressive symptoms in mice challenged with systemic LPS [63]. Therefore, targeting peripheral levels of monocyte-associated TNFα could be therapeutic in the treatment of depression.

In the CNS, IL-1β acts as a pleiotropic cytokine that activates both CNS microglia and astrocytes, propagating neuroinflammation. As with earlier cytokine studies linked to psychiatric illnesses, much of the data were collected from patients exhibiting depression secondary to an existing neuropathological state. Clinical analysis of MS patients showed increased circulating levels of IL-1β in patients that exhibited frequent and severe depressive episodes [64]. In rats, there is a behavioral-stress-associated increase in circulatory IL-1β, breakdown of the blood-brain barrier and severe neuroinflammation [65]. Laumet et al. implemented a murine model to show the role of IL-1 signaling in the progression of depression-like behavior through the regulation of tryptophan-kynurenine (KYN) [66]. Notably, the enzyme indoleamine 2,3-dioxygenase (IDO), stimulated by M1 macrophage-associated cytokines, dysregulates neurotransmission exhibited in depression. Increased IDO activity may cause both tryptophan depletion and increased neurotoxic metabolites of the kynurenine pathway, two alterations that have been hypothesized to cause depression. IDO regulates the metabolism of kynurenine from tryptophan, and downstream metabolism of KYN leads to conversion of KYN into QA by an enzyme known as kynurenine-monooxygenase (KMO) [67,68]. Laumet noted the elevated neuronal KMO was dependent on IL-1β availability and that the enzymatic activity of KMO on KYN was involved in the observed depression-like behavior [66]. IL-1β is a pyrogen that regulates cross-talk amongst CNS glial cells and immune compartments, thus making it a potential physiological target in the treatment of CNS disease [69]. 

## 4. The Inflammasome

Nod-like receptors (NLRs) are a family of innate cytosolic sensors responsible for the activation and assembly of inflammasome complexes [70]. Many components of the inflammasome act as typical PRRs, which can directly detect the presence of PAMPs [71]. However, more recently, components that form the Nod-like receptor pyrin (PYR) 3-containing (NLRP3) inflammasome, which do not respond to traditional DAMPs and PAMPs, respond to what are referred to as homeostasis-altering molecular processes (HAMPs), which allow the inflammasome to detect perturbations in cellular homeostasis [72,73]. While it remains unclear what ultimately activates these inflammasomes, this ability to indirectly recognize the presence of HAMPs is thought to provide an additional avenue for the recognition of pathogens that evade traditional detection by PRRs. However, it is becoming increasingly clear that these inflammasomes underlie many chronic inflammatory diseases resulting from sterile inflammation (inflammation in the absence of infection). Among others, the NLRP3 inflammasome can be activated by extracellular ATP released from dying cells, as well as in the presence of crystals, including uric acid crystals, cholesterol crystals and amyloid beta crystals underlying the development of gout, atherosclerosis and Alzheimer’s disease, respectively [74,75,76,77].

**Figure 3:**
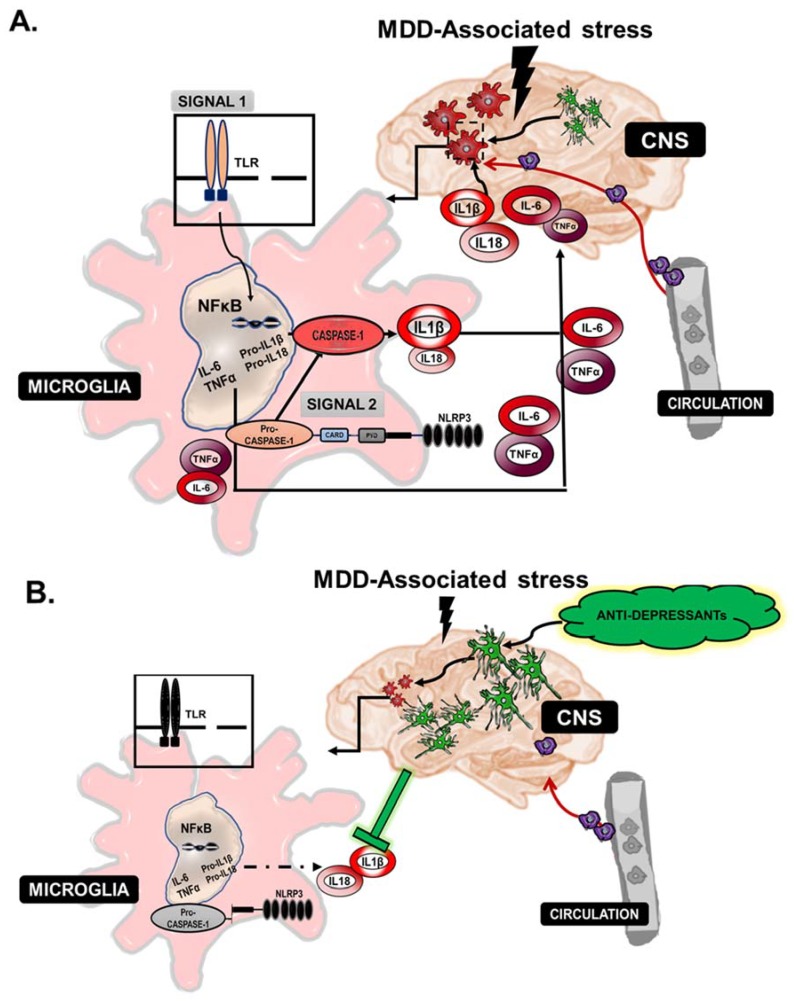
Macrophage-associated inflammasome activation in MDD. (**A**) The NLRP3 inflammasome complex is activated following a stress stimulus, which engages two inflammatory signals. The first signal is the activation of a family of innate membrane receptors, Toll-like receptors (TLRs), which lead to the activation of the canonical inflammatory NFκB pathway. This signal leads to the transcription of cytokines IL-1β and IL-18, while the inflammasome complex promotes the cleavage of IL-1β and IL-18 through the activation of caspase-1. In a pro-inflammatory state, circulating monocytes contribute to the expansion of M1 activation and propagation of increased IL-1β, IL-6 and TNFα, which act in a positive feedback loop. (**B**) Anti-depressant or ‘antagonist’ treatments foster the expansion or polarization of M2-microglia and consequent inhibition of M1-macropahges, lower levels of NLRP3 activation and eventually the attenuation of pro-inflammatory cytokine secretion in the circulation. Altogether, an increased M2 macrophage population and/or activation hinders the pro-inflammatory cytokine feedback loop, thus reducing anxiety and depressive-like behavior.

Of the inflammasome complexes discovered, NLRP1 and NLRP3 have been studied in neurological disorders [78]. The NLRP3 inflammasome complex is activated following a stress stimulus, which engages two distinct inflammatory signals (Figure 3) [71,79]. The first signal, for example, can be the activation of PRRs and the NFκB pathway. This signal promotes the transcription of cytokines IL1β and IL18, as well as NLRP3 itself, while the second signal promotes the assembly of the NLRP3 inflammasome. The inflammasome promotes the caspase-1-dependent cleavage of pro-IL-1β and pro-IL-18 into their mature or active forms. Emerging studies now focus heavily on understanding the mechanisms underlying NLRP3 activation in microglia in inflammation associated with neuropathology ranging from neurodegenerative diseases such as Alzheimer’s disease to psychiatric illnesses. Zhang et al. implemented a murine model of systemic inflammation with LPS administration in conjunction with a forced swim-test and demonstrated initiation of inflammasome activation correlated with depression symptomology. Whole mouse brains were analyzed for inflammasome components, and significant increases in protein expression of NLRP3 and caspase-1 were observed. In a separate murine model of chronic unpredictable mild stress (CUMS)-induced depression, Su et al. showed that the NLRP3 inflammasome mediated CUMS-induced depression-like behavior and that NLRP3 induction was associated with induction of NFκB protein activation [30]. Yue et al. observed a correlation between the purinergic receptor P2X7R, which promotes NLRP3 activation in response to extracellular ATP, and NLRP3 inflammasome activation in CNS-specific macrophages and showed that microglial activation correlated with the observed depressive symptoms [31]. P2X7A antagonist treatment lowered levels of NLRP3 and attenuated anxiety and depressive-like behaviors in Sprague-Dawley rats. Iwata et al. demonstrated that ‘psychological stress’ elicits extracellular ATP accumulation, which is a potential stimulus for the activation of P2X7R [32]. Clinical studies from patient populations have shed light on the significance of microglial inflammasome activation in MDD. In a cohort of untreated MDD patients versus those treated with anti-depressant amitriptyline, Alcocer-Gomez et al. observed increased circulating levels of IL-1β and IL-18 with increased red blood cell(RBC) expression of NLRP3 in the untreated population [40]. Associated with inflammasome activation, these patients exhibited increased macrophage-mediated ROS production, which perpetuated a positive feedback loop for NLRP3 activation. Thus, inflammasome assembly, caspase-1, IL-1 or the IL-1 receptor could be viable drug targets for the treatment of MDD.

## 5. Chronic Inflammation and Links to Depression

Humans exhibit various forms physiological and evolutionary stressors that manifest in chronic inflammation including, but not limited to, pregnancy, lifestyle, socio-economic stress and obesity. Emerging clinical research has linked inflammatory dysregulation and inflammation in the CNS to depressive disorder in humans, as well as in mice. Cepeda et al. collected data from the NHANES study and implemented a general population-based cross-sectional study (from 2007–2012), which demonstrated that increased inflammation, indicated by high levels of the inflammatory marker C-reactive protein (CRP), was associated with depression. From the 2009–2010 NHANES survey, Rethorst et al. found elevated rates of inflammation in individuals with depression above the age of 18. Pasco et al. presented a retrospective cohort study of 1494 randomly-selected women and found a 44% increase in the hazards ratio for depression for every standard deviation increase in high-sensitivity CRP levels in the blood [80].

Obesity incidences are on the rise, and obesity-associated diseases are an economic and global concern. Clinically, obesity is a significant risk factor for developing depression, and meta-analyses support the co-occurrence of obesity and psychiatric illnesses, specifically mood disorders [81,82]. Individuals with increased body mass index (BMI) associated with increased adiposity were 30% more likely to exhibit clinical symptoms of depression [82]. Studies compiled from the National Health and Nutrition Examination Survey (NHANES) indicate that obesity and being overweight lead to depression and anxiety in American women [83]. Randomized control studies in elderly individuals with metabolic syndrome showed the association of elevated depressive moods [84]. 

Obesity stimulates inflammatory processes, and the persistent nature of this condition leads to uncontrolled inflammation, primarily mediated by macrophages. Tissue-resident macrophages, specifically adipose tissue macrophages (ATMs), are implicated in obesity-associated systemic inflammation [85,86]. Over-nutrition-associated metabolic stress leads to ATM remodeling, as triglycerides accumulate in adipocytes and lead to adipocyte hyperplasia and hypertrophy [87]. Cellular stress is compounded by severe tissue hypoxia, increased ROS production, which promotes the formation of “crown-like structures” composed of necrotic adipocytes surrounded by monocyte-derived M1 macrophages. Macrophage heterogeneity is critical to regulating the progression of obesity-induced immune dysregulation [88]. The production of pro-inflammatory cytokines by ATMs has been linked to insulin resistance and the chronic inflammation associated with obesity [25]. These pro-inflammatory factors provide chemical cues for infiltration and activation of resident immune cells, which perpetuate inflammation in adipose and hepatic tissues, leading to insulin resistance.

Studies in mice and men have shown that obesity potentiates inflammatory cytokine expression in multiple regions of the brain, as well as the entry of circulating monocytes, which contribute to cytokine trafficking across the BBB in sufficient levels to initiate immune dysfunction [17]. Trafficked monocytes contribute to the M1 macrophage population in the CNS and perpetuate the accumulation of IL-6 and IL-1β in the CNS parenchyma [39]. In order to study the role of macrophage polarization in obesity-associated neuroinflammation, we utilized mice with a targeted deletion in the recepteur d’origine nantais (Ron) receptor. Ron is a receptor tyrosine kinase that is expressed on tissue-resident macrophages including ATMs, Kupffer cells and microglia [89,90]. The ligand for the Ron receptor is the macrophage-stimulating protein (MSP), which is produced primarily in the liver and circulates in an inactive form. MSP is cleaved to its active form upon entry into inflammatory tissues by a variety of proteases. Ligand-dependent activation of Ron in vitro downregulates PRR-induced NF-κB activity and M1 polarization and promotes an M2 macrophage phenotype associated with elevated levels of Arg-1 expression. The in vivo deletion of the ligand-binding domain of Ron limits reparative M2 macrophage activation and exacerbates diet-induced obesity and associated hepatic inflammation [89]. We have recently shown that chronic restraint of M2 macrophage activation due to the absence of Ron results in M1-associated neuroinflammation and increased levels of metabolic stress in the CNS. In the context of the aggressive demyelinating disease induced in the murine model of multiple sclerosis (MS), murine experimental autoimmune encephalomyelitis (EAE), impaired M2 macrophage activation in the absence of Ron leads to increased MS pathology [90]. In a model of high-fat diet-induced obesity, a lack of Ron results in severe neuroinflammation associated with decreased expression of the M2 marker Arg-1 and increased M1 markers IL-1β, IL-6 and TNFα when compared to WT control animals [90]. In unpublished studies, we have also shown that Ron inhibits the NLRP3 inflammasome in cultured microglia, and mice lacking Ron exhibit elevated NLRP3 inflammasome activation in the hippocampus in the context of diet-induced obesity. Thus, Ron plays a protective role in CNS inflammation, at least in part, by regulating macrophage polarization and inflammasome activation. Regulating the balance between M1 and M2 microglial states could therefore provide a potential therapeutic strategy in the treatment of a spectrum of neural disorders with chronic inflammation as an underlying cause.

## 6. Drug Therapeutics Targeting Macrophage Polarization

In a healthy brain, the microglia are in a ‘quiescent’ or alternatively-activated (M2) state [19,25,91]. In neurogenerative diseases and aging-associated dementia, M2-microglia have been implicated in wound healing and restoration of health through inhibition of inflammatory cytokines [91]. An emerging therapeutic strategy is targeting M2-macrophage expansion in inflamed tissue niches to counter uncontrolled inflammation perpetrated by M1-macrophages. In a relapsing and remitting murine model of MS, myelin oligodendrocyte glycoprotein (MOG)-immunized mice exhibited increased M2-microglial expansion immediately preceding remission [19,92]. Therefore, it does not come as a surprise that a spectrum of drugs targeting psychological disorders is targeting the expansion and maintenance of the M2-microglial population. The peroxisome proliferator-activated receptor gamma (PPARγ) agonist from the thiazolidinediones family, pioglitazone (PIO), inhibits the IL-6 pathway in mice. Liao et al. demonstrated an improvement in depressive behaviors induced by intraventricular administration of LPS in mice, associated with a decrease in IL-6 and restoration in BDNF levels [55]. The SSRIs, paroxetine and sertraline, have a direct suppressive effect on microglial activation and pro-inflammatory cytokine secretion in mice [93,94]. Talmon et al. demonstrated a significant role of alternatively-activated M2-macrophages and its anti-inflammatory effects in the treatment of depressive patients [95]. In patients taking the prescription drug vortioxetine, an antioxidant, drug treatment stimulated a phorbol 12-myristate 13-acetate (PMA)-induced oxidative burst in monocytes, which polarized the cells from the M1 to the M2 phenotype [95]. Another group of drugs, nonsteroidal anti-inflammatory drugs (NSAIDs), such as ibuprofen, are oftentimes used in combination with antidepressants. NSAIDs target cyclooxygenase-2 (Cox-2), which is a potentiator of M1 macrophage activation, and Cox-2 has been used to target the accumulation of inflammatory cytokines. Clinical trials that incorporated drugs and assessment scales with MDD patients found that supplementation of anti-depressants with a selective nonsteroidal anti-inflammatory Cox-2 inhibitor, celecoxib, significantly improved the Hamilton Depression Rating Scale (HDRS) scores [96]. Kohler et al. compiled a comprehensive meta-analysis of 14-trials that either evaluated cytokine inhibitors or NSAIDs and found a pooled effect estimate of anti-inflammatory treatment-associated reduction of depressive symptoms compared with placebo [96,97]. In this meta-analysis, NSAIDS were used as mono-therapies in addition to add-on treatments with SSRIs in a general population of roughly 6000 patients [96]. To date, these studies are the most convincing clinical-meta analyses to confirm the positive effect of anti-inflammatory treatment in treating depression. Cytokine-associated inflammation in MDD stimulates monoamine oxidation, which signals uncontrolled ROS accumulation and lipid peroxidation. In a cohort of MDD patients versus healthy controls, Bilici et al. found that patients with depression exhibited elevated levels of lipid peroxidation [98]. A short-term SSRI treatment in depressive individuals in this study had a striking suppressive effect on lipid peroxidation and systemic inflammation. 

Lipid mediators are another class of anti-inflammatory agents that support M2 polarization. Pro-resolving molecules such as resolvin D2 and lipoxin A4 have been implicated in the maintenance of M2 microglia and polarization of M1-macrophage towards an M2-macrophage phenotype [99]. Studies by Deyama et al. found that the anti-depressant effect of lipid mediators resolvin D1 and resolvin D2 and their downstream target, mammalian target of rapamycin complex 1 signaling (mTOR) pathway, reversed LPS-induced depressive behaviors in mice [100]. The prior generation of anti-depressant therapies has focused on surface receptor signaling mechanisms for immediate symptom management; however, evolving research is focusing on the long-term adaptations in cellular signaling mechanisms for sustained antidepressant drug therapy. Emerging studies have identified the transcription factor cyclic AMP response element binding protein (CREB) as a key cellular player in the etiology of MDD treatment and that CREB expression is decreased in depressive pathophysiology [101]. Circulating catecholamines and eicosanoids, such as prostaglandin E2 (PGE2) induce anti-inflammatory M2 macrophages through the induction of the cAMP signaling pathway. Specifically, PGE2 stimulates M2 polarization through CREB-mediated activation of Krüppel-like factor 4 (KLF4). CREB-enhancing anti-depressants are not prominently available; therefore, targeting this cAMP and CREB pathways is a promising avenue for chronic anti-inflammatory therapeutics for MDD [102]. BDNF is a downstream effector of CREB and has been shown to mitigate and repair depression-associated neuronal injury [102]. In all cases, targeting the heterogenic balance between M1 and M2 microglia in the CNS is a potential strategy for curbing the inflammation-induced cytokine secretion associated with MDD symptoms

## 7. Summary

Emerging studies in neuropsychiatric illnesses and MDD highlight the importance of studying the immunological processes underlying MDD. Macrophage heterogeneity and uncontrolled cytokine-associated inflammation have been well characterized in neurodegenerative diseases, but a more comprehensive understanding of macrophage-mediated neuropathology in MDD is still needed. A ‘macrophage theory of depression’ has been proposed, whereby uncontrolled inflammation is supported by macrophage activation and increased secretion of inflammatory cytokines into the CNS proper. A multitude of studies has confirmed the positive effects of anti-depressants on macrophage-associated cytokine production levels. The CNS and peripheral accumulation of cytokines such as IL-6, TNF-α and IL-1β perpetuates activation of microglial cells that support the positive inflammatory loop. Limiting the focal production of cytokines in addition to targeting direct cellular pathways is likely to be key in future drug development. With any and all disease states that sustain chronic inflammation, the interactive relationships between environmental and genetic stressors likely play a significant role in determining individual responses to anti-depressants. Therefore, genomic studies in large cohorts of MDD patients would illuminate a variety of genetic tools that may help in understanding how to regulate predisposition to inflammatory stress in the CNS.
